# Association of Robson Ten Group Classification System with neonatal/postneonatal mortality: an analysis for the effect of the mass migration

**DOI:** 10.1016/j.xagr.2025.100464

**Published:** 2025-02-21

**Authors:** Damla Çarkçı Yıldız, Elif Gül Yapar Eyi

**Affiliations:** University of Health Sciences, Ministry of Health Ankara Bilkent City Hospital, Çankaya Ankara Türkiye

**Keywords:** Robson classification, cesarean section, neonatal mortality, postneonatal mortality, Türkiye, refugees, asylum seekers

## Abstract

**Background:**

Türkiye is the largest host country for refugees and asylum seekers and provides comprehensive maternal care, including antenatal, intrapartum, and postpartum services. However, there is a notable gap in comparative data regarding neonatal and postneonatal mortality for cesarean sections among Turkish citizens and refugees/asylum seekers.

**Objective:**

The study aims to: (1) Compare neonatal and postneonatal mortality rates across Robson groups among Turkish citizens and refugees/asylum seekers. (2) Identify risk factors related to neonatal and postneonatal deaths, considering Robson Classification, clinical obstetric parameters, and demographics. (3) Investigate the primary causes of neonatal/postneonatal deaths in a hospital with a cesarean section rate of 48.43%.

**Study Design:**

A retrospective cross-sectional study based on hospital electronic data was conducted, analyzing 25,631 cesarean section births. The participants included 89% Turkish citizens and 11% refugees/asylum seekers. Student's t-test, χ² -test, Mann-Whitney U test, Kruskal-Wallis analysis, and logistic regression were applied to identify risk factors and make comparisons.

**Results:**

Neonatal Deaths: Out of 26,474 live births, 513 newborns died. Mortality rates were 21.94 per 1000 live births for refugees/asylum seekers and 19.05 per 1000 live births for Turkish citizens. Robson Group Distribution: The distribution of cesarean births across Robson groups was varied, with the highest mortality rates observed in groups R8, R9, R10, R6, and R7 (*p*=.001). Risk Factors: Logistic regression analysis identified Robson groups, fetal presentation, gestational age, Apgar scores, and newborn weight/height as significant risk factors for neonatal /postneonatal mortality. However, no significant associations were found with demographic factors, including maternal age, parity, and nationality. Main Causes of Deaths: The leading causes were prematurity (452 cases), congenital abnormalities (160 cases), infections (78 cases), asphyxia (17 cases), and meconium aspiration syndrome (9 cases).

**Conclusion:**

The integration of the Robson Classification with neonatal and postneonatal mortality data offers a structured method to assess cesarean section outcomes, emphasizing the significant variation in mortality rates across different Robson groups. Notably, the highest risks were linked to multiple pregnancies, abnormal fetal presentations, and preterm births.

**AJOG Global Reports at a Glance**

**Why was this study conducted?**

Intrapartum-related mortality and/or perinatal mortality have been documented in the cesarean model analyses, yet no study has reported neonatal/postneonatal mortality across Robson groups and maternal demographics (age, nationality, parity).

**Key findings**

A total of 513 neonatal/postneonatal deaths occurred in 25,631 cesarean sections that were performed on Turkish citizens: 89.00% and refugees/asylum seekers: 11.00%. Key risk factors for neonatal/postneonatal deaths identified through logistic regression analyses were: Robson groups, fetal presentation, Apgar scores, birthweight, and height. Maternal age, nationality, and parity were not among the risk factors for neonatal/postneonatal deaths under standard obstetric and neonatal care.

**What does this add to what is known?**

Integrating Robson groups with neonatal and postneonatal mortality enables obstetricians and perinatologists to move beyond traditional cesarean section audits toward a more holistic perinatal care strategy to underscore the need for tailored obstetric and neonatal interventions.

## Introduction

In Türkiye, the rate of cesarean births has exceeded that of vaginal deliveries,^1^, ^2^, ^3^, ^4^ leading to ongoing initiatives aimed at reducing cesarean section (CS) rates. In response, the Ministry of Health of Türkiye adopted the Robson 10-Group Classification System (R TGCS)^5^^,^^6^ in 2012 to systematically monitor and analyze CS rates,^7^^,^^8^ ensuring alignment with global standards set by the World Health Organization (WHO),^9^ the International Federation of Gynecology and Obstetrics,^10^ and the European Board of Obstetrics and Gynecology.^11^, ^12^ In 2014, Türkiye implemented an electronic registration system to systematically document all cesarean births based on the R TGCS, a system that remains in use today. Additionally, a separate electronic death registration system is employed to record stillbirths, as well as inpatient neonatal and postneonatal deaths, for cases involving infants weighing over 500 grams or beyond 22 weeks of gestation.^12^

Rather than conducting discrete analyses, integrating neonatal and postneonatal mortality data with the R TGCS could offer a more comprehensive perspective on obstetric outcomes, potentially bridging the gap between obstetric decision-making and neonatal care. This integrated approach would enable obstetricians/perinatologists to move beyond traditional cesarean section audits, facilitating a more holistic perinatal care strategy, early risk identification, and a deeper understanding of neonatal/postneonatal outcomes.

Türkiye has been hosting a substantial number of refugees and asylum seekers, with 3,789,935 registered individuals in 2023.^13^ Among them, approximately one million were between the ages of 15 and 44, with 40% being female.^13^^,^^14^ The electronic registration systems also document cesarean deliveries, RTGCS categorizations, and neonatal and postneonatal mortality among refugee and asylum-seeker populations.

This study aims to:

• Assess and compare neonatal and postneonatal mortality rates across the R TGCS among Turkish (TR) citizens and refugee/asylum-seekers.
• Investigate risk factors associated with neonatal and postneonatal mortality based on R TGCS, maternal demographics, and the leading causes of death in a tertiary referral center.

## Materials and methods

### Ethical approval

This retrospective cross-sectional study was conducted at Ankara Bilkent City Hospital between September 2019 and January 2023. Ethical approval was granted by the Clinical Research Ethics Committee (Approval No: E2-23-3725, dated 26.05.2023). Informed consent was not required, as the study utilized aggregated birth registry and neonatal/postneonatal mortality data from the Health Integrated Campus of the hospital; however, all women provided consent for admission and medical care if they were conscious. The study adhered to the ethical principles outlined in the Declaration of Helsinki.

### Study population and design

During the study period, a total of 52,926 live births were recorded, with 25,631 women (48.43%) delivering via cesarean section, all of whom were included in the study. Among them, 22,812 (89.00%) were TR citizens, while 2,819 (11.00%) were refugees or asylum seekers. The refugee population primarily comprised individuals from:

• Syria (n = 2,021, 71.7%)
• Iraq (n = 420, 14.9%)
• Afghanistan (n = 110, 3.9%)
• Azerbaijan (n = 54, 1.9%)
• Other countries (n = 204, 7.6%)

A total of 25,537 women were classified according to the R TGCS.^7^^,^^8^

### Definitions

We applied the WHO definition of miscarriage, setting the threshold at <22 weeks of gestation and/or birth weight <500 grams, as the 50th percentile fetal weight at 20 weeks is 325 to 350 grams.^15^ Neonatal mortality was defined as deaths occurring at ≥ 22 weeks and/or ≥ 500 grams. Neonatal mortality, expressed per 1000 live births, was further categorized as follows:

• Early neonatal mortality: Deaths within the first 0 to 6 days.
• Late neonatal mortality: Deaths between 7 and 28 days.

Postneonatal mortality referred to in-hospital deaths occurring after 28 days.^12^^,^^15^

Outcome

Primary Outcome: was to assess the inpatient neonatal/postneonatal mortality following CS.

Secondary Outcomes: included the identification of the risk factors for neonatal and postneonatal mortality in terms of:

• Maternal demographics: nationality, age, gravidity, parity, number of living children.
• Newborn characteristics: Gestational age, 1st and 5th-minute Apgar scores, gender, birth measurements (height, weight).
• Causes of neonatal/postneonatal mortality: Prematurity/immaturity, Respiratory distress syndrome (RDS), congenital anomalies, infections, perinatal asphyxia, Meconium aspiration syndrome (MAS), and others.

### Statistical analysis

Statistical analysis was performed using IBM SPSS Statistics for Windows, Version 22.0 (IBM Corp., Armonk, NY). Categorical variables were presented as numbers (percentages or per 1,000 live births) and compared using the χ² or Fisher's exact test. Continuous variables were expressed as mean/median ± standard deviation (SD) and analyzed using the Student's *t*-test or Mann-Whitney *U* test for non-normally distributed data. Data normality was assessed graphically and with the Shapiro-Wilk test.

For 2-group comparisons, the Mann-Whitney *U* test was applied, while the Kruskal-Wallis test was used for comparisons involving more than 2 groups. Bonferroni correction was performed for multiple pairwise comparisons. A *P*-value of <.05 was considered statistically significant.

Logistic regression analysis was conducted to identify risk factors for neonatal and postneonatal mortality. Results were reported as odds ratios (OR) with 95% confidence intervals (CI) for RTGCS, maternal demographics, and neonatal risk factors.

## Results

A total of 26,474 neonates: 24,691 singletons and 876 multiples **(**846 twins, 29 triplets, and one quadruplet) were delivered via 25,631 CSs.

### R-TGCS distribution

**Figure 1:**
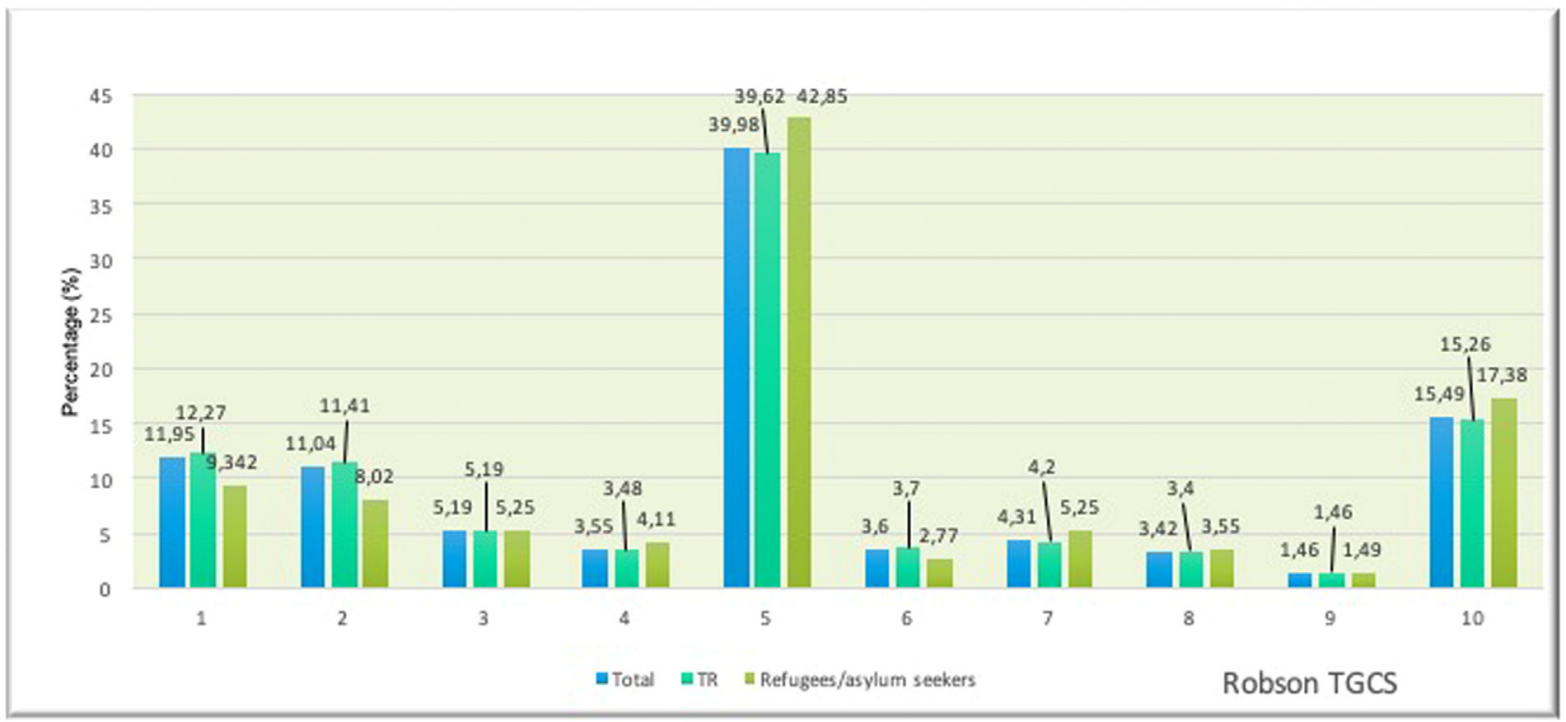
Distribution of the 25,631 CSs according to Robson TGCS as total, in TR citizens and refugees/ asylum seekers

The R-TGCS distribution for the entire study group was as follows (Table 1):

• R1: 11.95% (3062/25,631)
• R2: 11.04% (2829/25,631)
• R3: 5.19% (1331/25,631)
• R4: 3.55% (911/25,631)
• R5: 39.98% (10,247/25,631)
• R6: 3.60% (923/25,631)
• R7: 4.30% (1105/25,631)
• R8: 3.42% (876/25,631)
• R9: 1.46% (376/25,631)
• R10: 15.49% (3971/25,631)
• R5 (previous CS) was the most dominant category across all years, comprising 39.62% of TR citizens and 42.85% of refugees/asylum seekers. The CS rate was significantly higher in refugees/asylum seekers (*P*<.001).
• R10 (preterm births), R1 (nulliparous, term, spontaneous labor), and R2 (nulliparous, term, induced/CS before labor) were also major contributors to overall CS rates (Figure 1).

Damla Çarkçı Yıldız. Association of Robson Ten Group Classification System with neonatal/postneonatal mortality. AJOG Glob Rep 2024.
• R9 (transverse/oblique lie) remained the smallest category, with a similar distribution between TR citizens (1.46%) and refugees/asylum seekers (1.49%).
• Significant differences were observed in R1, R2, R5, R6, R7, and R10 between TR citizens and refugees/asylum seekers (*P*<.001), suggesting:
  ○ Higher nulliparity rates among TR citizens (R1, R2).
  ○ Higher multiparity and preterm birth rates among refugees/asylum seekers (*P*<.001) (Table 1).

**Table 1:** Distribution of the Robson groups according to nationality between September 2019 and January 2023

		Total		TR citizens		Refugees/asylum seekers		
Robson group	Group characteristics	n	%	n	%	N	%	*P*^a^
1	Nulliparous women with single cephalic pregnancy, ≥37 wk gestation in spontaneous labor	3062	11.95	2799	12.27	263	9.32	<.001
2	Nulliparous women with single cephalic pregnancy, ≥37 wk gestation who either had labor induced or were delivered by CS before labor	2829	11.04	2603	11.41	226	8.02	<.001
3	Multiparous women without a previous uterine scar, with single cephalic pregnancy, ≥37 wk gestation in spontaneous labor	1331	5.19	1183	5.19	148	5.25	.92
4	Multiparous women without a previous uterine scar, with single cephalic pregnancy, ≥37 wk gestation who either had labor induced or were delivered by CS before labor	911	3.55	795	3.48	116	4.11	.09
5	All multiparous women with at least one previous uterine scar, with single cephalic pregnancy, ≥37 wk gestation	10,247	39.98	9039	39.62	1208	42.85	.001*
6	All nulliparous women with a single breech pregnancy	923	3.60	845	3.70	78	2.77	.01*
7	All multiparous women with a single breech pregnancy, including women with previous uterine scars	1105	4.31	957	4.20	148	5.25	.01*
8	All women with multiple pregnancies, including women with previous uterine scars	876	3.42	776	3.40	100	3.55	.72
9	All women with a single pregnancy with a transverse or oblique lie, including women with previous uterine scars	376	1.46	334	1.46	42	1.49	.98
10	All single, cephalic, < 37 wk (including previous CS)	3971	15.49	3481	15.26	490	17.38	.003*
Total		25,631	100	22,812	100	2804	100	

a Chi-square analysis was used for comparison between groups.

### Maternal demographics

• The mean maternal age was 29.19±5.68 years, with 0.36% under 18 years (Table 2).

**Table 2:** Maternal demographics for CS births according to nationality and Robson TGCS

		Total (n=25,631)		TR citizens (n=22,812)		Refugees/asylum seekers (n=2819)		*P*
**Maternal age (years) Mean± SD**		29,19±5,68		29.41±5.57		27.39±6.19		**<.001^a^**
**Gravidity**		2.57±1.92		2.51±1.44		3.17±1.91		**<.001 ^a^**
		**N**	**%**	**N**	**%**	**N**	**%**	
**Age categories**	<18	92	.34	34	.15	58	2.1	**<.001 ^b^**
	18 to 26	7 243	28.26	6 094	28.91	1149	40.76	**<.001 ^b^**
	26 to 35	14 400	56.38	13 116	57.50	1335	47.36	**<.001 ^b^**
	≥35	3 830	15.00	3 568	15.64	277	9.83	**<.001 ^b^**
^a^Mann Whitney U test								
^b^Chi-square analysis.								

		Age mean ± SD	Gravidity	Parity	Living children	*P*		
**Robson**	1	26.32±5.06	1.26±0.76	.00±.00	.00±.00	**<.001**		
	2	25.77±4.55	1.24±0.61	.00±.00	.00±.00			
	3	30.99±5.77	3.27±1.43	1.80±1.18	1.71±1.05			
	4	31.48±5.81	3.40±1.53	1.82±1.13	1.69±1.19			
	5	30.35±5.21	3.14±1.30	1.68±0.92	1.62±0.92			
	6	26.02±4.97	1.31±0.75	.00±.00	.00±.00			
	7	30.97±5.70	3.29±1.42	1.80±1.07	1.69±1.04			
	8	29.53±5.64	2.45±1.51	1.05±1.18	0.87±1.30			
	9	29.85±6.13	2.75±1.70	1.19±1.25	0.99±1.35			
	10	29.70±5.96	2.86±1.62	1.38±1.19	1.24±1.20			
^b^Kruskal-Wallis analysis *P*<.001.
• Age distribution: 28.26% were aged 18 to 26 years, 56.38% were 26 to 35 years, and 15.00% were >35 years.
  ○ Maternal age varied significantly across R-TGCS categories and by nationality (*P*<.001).
  ○ The oldest mothers were in R4**,** while the youngest were in R2 (*P*<.001).
  ○ Refugees/asylum seekers were younger than TR citizens (*P*<.001).
• The mean values for gravidity, parity, number of living children, and gestational age at birth were:
  ○ Gravidity: 2.59±1.51
  ○ Parity: 1.17±1.15
  ○ Number of living children: 1.10±1.14
  ○ Gestational age at birth: 37.42±23.68 weeks
• Gravidity, parity, and the number of living children were significantly higher among refugees/asylum seekers than TR citizens, with significant differences across R-TGCS categories (*P*<.001)

### Fetal and newborn characteristics

• Cephalic, breech, and transverse-oblique presentations constituted 89.00%, 9.01%, and 1.99% of the study group, respectively, depicting significant differences between groups (*p*<.001).
• Gestational age analysis revealed that R 2 had the highest and R 8 had the lowest gestational age, with significant differences between the groups (*p*<.001).
• In 87.24% of the neonates, the 1st minute APGAR score was ≥ 7, whereas the 5th minute APGAR score was ≥ 7 in 96.80%. There were significant differences between nationalities in terms of 1st minute Apgar score, resulting from the values in the Apgar 4 to 6 and Apgar ≥ 7 categories (*P*<.001) (Table 3).

**Table 3:** Fetal and newborn characteristics according to nationality and R TGCS

		Fetal presentation^a^						Gestational age (week)^b^
		Vertex		Breech		Transverse Oblique		
		N	%	n	%	n	%	Mean ± SD
**Robson**	**1**	3062	13.0	0	0.0	0	0.0	38.89±2.01
	**2**	2829	12.0	0	0.0	0	0.0	39.22±1.96
	**3**	1331	5.6	0	0.0	0	0.0	38.88±8.73
	**4**	911	3.9	0	0.0	0	0.0	39.00±2.69
	**5**	10,247	43.5	0	0.0	0	0.0	38.45±37.62
	**6**	0	0.0	923	38.7	0	0.0	36.54±3.74
	**7**	0	0.0	1105	46.3	0	0.0	36.40±3.86
	**8**	1210	5.1	357	15.0	152	28.8	33.18±3.71
	**9**	0	0.0	0	0.0	376	71.2	35.60±4.70
	**10**	3971	16.9	0	0.0	0	0.0	33.99±3.23
^a^Chi-square analysis *P*<.001								
^b^Kruskal-Wallis analysis *P*<.001								

Apgar scores^a^		TR		Refugees/asylum seekers				
		N	%	n	%	P*		
**1st minute Apgar score**	0-3	505	2.14	64	2.19	<.001		
	4-6	2439	10.35	369	12.65			
	≥ 7	20,614	87.50	2483	85.15			
**5th minute Apgar score**	0-3	137	0.58	21	0.72	.653		
	4-6	615	2.61	75	2.57			
	≥ 7	22,806	96.81	2820	96.71			
*Chi-square analysis								

Distribution of the newborn gender, weight and height at birth^a^								
		**N**	**%**					
**Gender**	Female	12,544	47.38					
	Male	13,930	52.62					
**Height, Mean ± SD (cm)**^a^		48.88±3.63						
**Weight, Mean ± SD (grams)**^a^		3022.10±699.77						

Birthweight and height of the babies according to nationality^a^								
	TR	Refugees/asylum seekers						
	Mean + SD	Mean + SD	*P*^c^					
Height (cm)	48.90±3.64	48.70 ± 3.54	<.001					
Birthweight (grams)	3028.78±703.29	2968.15 ± 668.34	<.001					
^c^Mann-Whitney U test								
Newborn's height and weight according to Robson classification^b^								

		Height (cm)		Weight (grams)				
		Mean + SD	P*	Mean + SD	P*			
**Robson**	1	50.37±2.20	**<.001**	3359.12±548.69	<.001			
	2	50.17±1.88		3275.15±462.22				
	3	50.66±2.24		3454.25±600.45				
	4	50.32±2.06		3316.58±524.20				
	5	50.00±1.71		3233.25±413.78				
	6	47.71±4.43		2778.61±769.75				
	7	47.97±4.57		2860,24±801,05				
	8	43.80±4.44		2025.06±602.67				
	9	47.15±5.66		2711.57±968.03				
	10	45.88±4.55		2387.35±706.49				
^b^Kruskal-Wallis analysis								

Neonatal Unit Admission according to Robson TGCS^a^								
		Neonatal Unit Admission						
		Present		Absent				
		n	%	N	%	P*		
**Robson TGCS**	1	411	13.42	2651	86.58	<.001		
	2	306	10.82	2523	89.18			
	3	186	13.97	1145	86.03			
	4	99	10.87	812	89.13			
	5	951	9.28	9296	90.72			
	6	223	24.16	700	75.84			
	7	318	28.78	787	71.22			
	8	932	54.22	787	45.78			
	9	143	38.03	233	61.97			
	10	1974	49.71	1997	50.29			
Total		5543	20.94	20.931	79.06			
^a^Chi-square analysis *P*<.001
• There were 13,930 boys and 12,544 girls. R 5 (39.62% vs 42.83%) was the major and R 9 (1.46 % vs 1.49%) was the minor group for TR citizens and refugees/asylum seekers, respectively.

The baby height and weight of TR citizens were significantly higher than those of refugees/ asylum seekers (*P*<.001) Birthweight distribution was shown in Table 3. Babies were the tallest and heaviest in R 3 and shortest and lightest in R 8. Significant differences existed in baby height and weight among the R TGCS (*P*<.001).

The distribution of the neonatal 1st-minute and 5th-minute Apgar scores admitted to the neonatal unit according to R groups was depicted in Supplemental 1 according to nationality and statistically significant differences existed according to nationality.

### Neonatal and postneonatal mortality

A total of 513 neonatal and postneonatal deaths were documented (Supplemental 1).

Mortality rates were similar between refugees/ asylum seekers **(**21.94 per 1000 live births**)** and TR citizens **(**19.05 per 1,000 live births**)** (*P*=.28).

### Timing and distribution of deaths

**Figure 2:**
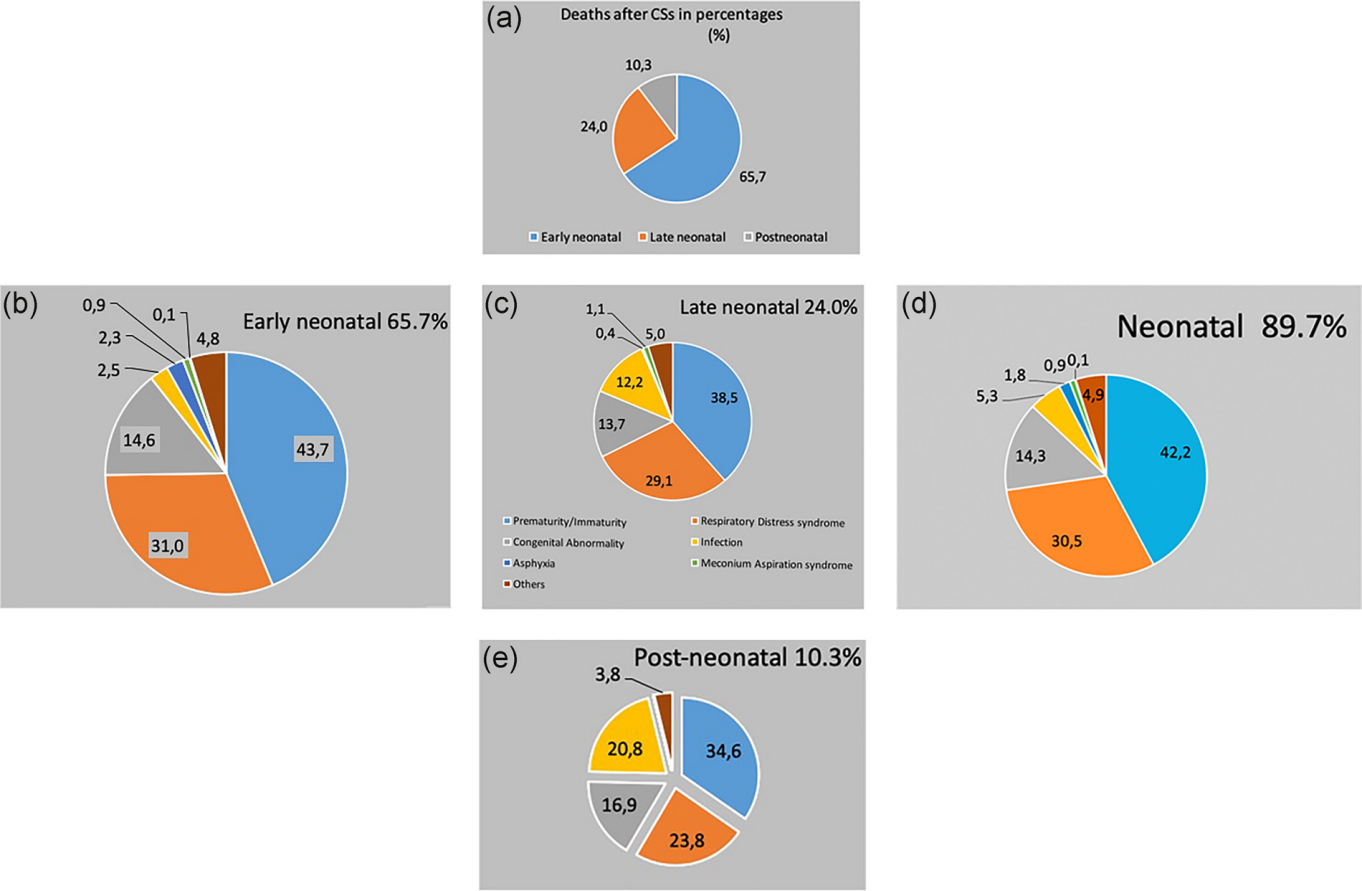
Distribution of 513 deaths according to the early neonatal, late neonatal, neonatal, and postneonatal period

**Figure 3:**
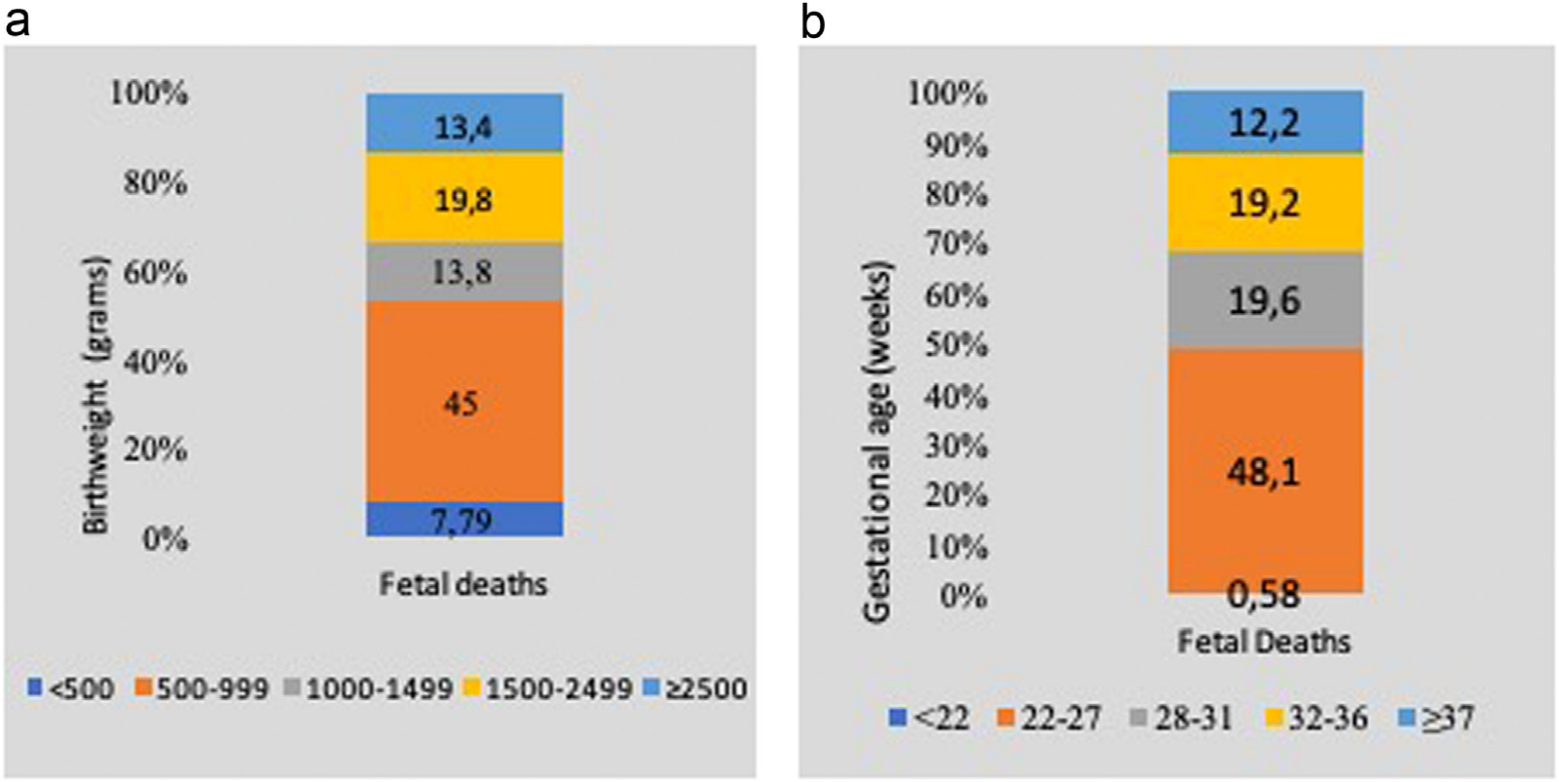
Birthweight distribution and gestational age of 513 neonatal/postneonatal deaths

**Figure 4:**
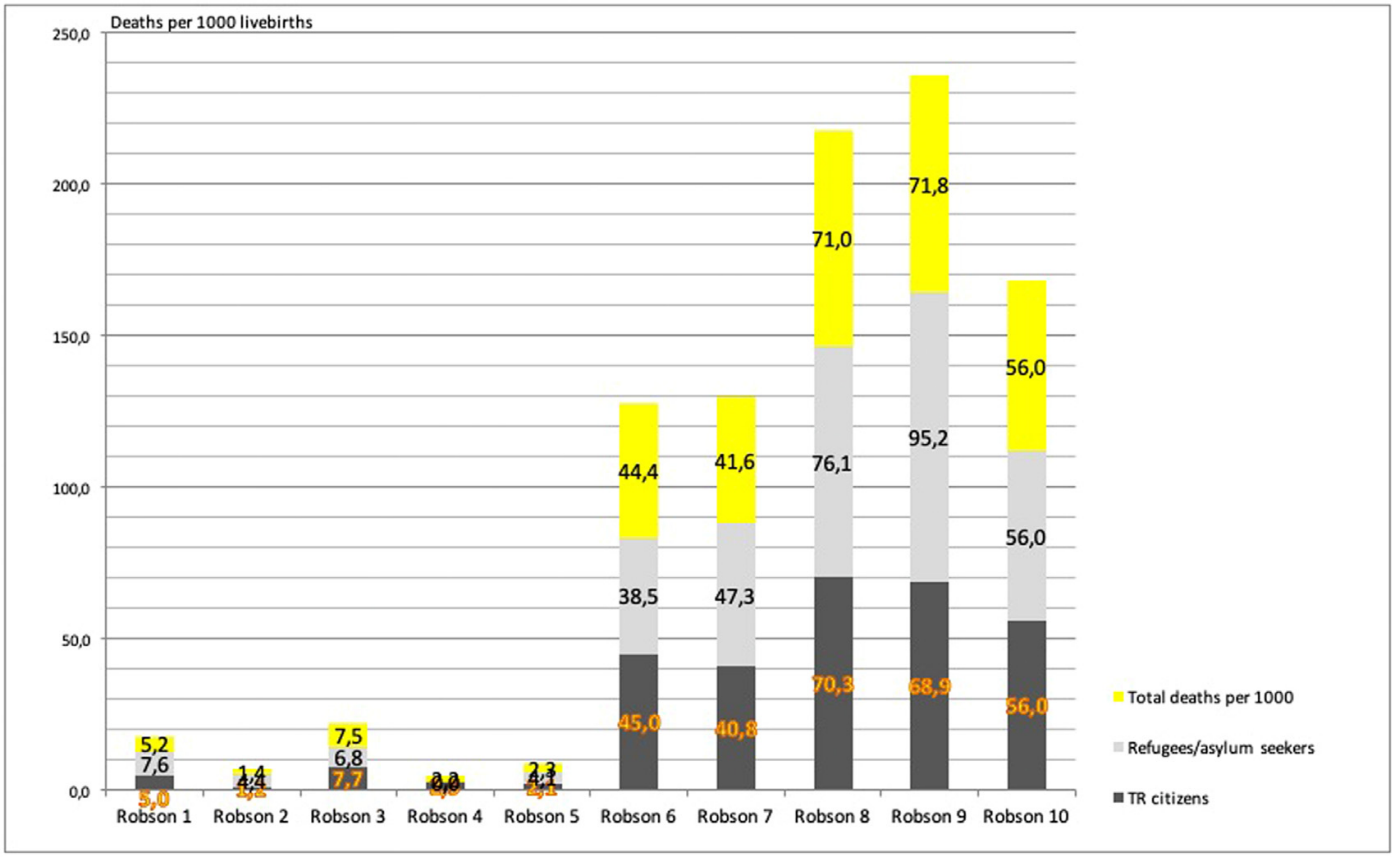
Distribution of neonatal/postneonatal deaths (per 1000 livebirths) according to nationality and Robson TGCS

Figures 2, 3, and 4 present:

• Timing of deaths
• Birth weight and gestational age
• Mortality distribution by R-TGCS and nationality, respectively.

Damla Çarkçı Yıldız. Association of Robson Ten Group Classification System with neonatal/postneonatal mortality. AJOG Glob Rep 2024.

Damla Çarkçı Yıldız. Association of Robson Ten Group Classification System with neonatal/postneonatal mortality. AJOG Glob Rep 2024.

Damla Çarkçı Yıldız. Association of Robson Ten Group Classification System with neonatal/postneonatal mortality. AJOG Glob Rep 2024.

Highest mortality rates**:**

○ R9 (transverse/oblique lie)
○ R8 (multiple pregnancies)
○ R10 (preterm births)
  • Lowest mortality rate**:** R2 (Figure 4).

Significant differences were detected among R-TGCS groups (*P*<.001), particularly between:

• R1 vs R6, R7, R8, R9, R10
• R2 vs R6, R7, R8, R9, R10
• R3 vs R5, R6, R7, R8, R9, R10
• R4 vs R6, R7, R8, R9, R10
• R5 vs R6, R7, R8, R9, R10 (Supplemental 2).

Variance analysis indicated a significant association between neonatal/postneonatal mortality and Robson groups, birth weight, birth length, Apgar scores, and gestational age, whereas nationality, maternal age, gravidity, and parity showed no significant correlation (Table 4).

**Table 4:** Risk factors for neonatal and postneonatal deaths

		B	SE	Wald	Sig.	Exp(B)	95% CI for EXP(B)	
							Lower	Upper
**Maternal variables**	Nationality	−0.151	0.172	0.769	0.380	0.860	0.614	1.204
	Age	−0.018	0.022	0.684	0.408	0.982	0.941	1.025
	Age category	0.285	0.188	2.306	0.129	1.330	0.921	1.920
	Gravida	−0.059	0.057	1.091	0.296	0.943	0.844	1.053
	Parity	−0.114	0.105	1.175	0.278	0.892	0.725	1.097
	Number of living children	0.149	0.093	2.579	0.108	1.160	0.968	1.391
	Robson	0.092	0.025	13.244	0.000	1.097	1.044	1.153
**Neonatal**	Gestational age	0.026	0.018	2.122	0.145	1.026	0.991	1.062
	Presentation	0.283	0.107	6.948	0.008	1.327	1.075	1.639
	1st-minute Apgar	0.121	0.094	1.664	0.197	1.129	0.939	1.357
	!st -minute Apgar category	0.460	0.209	4.839	0.028	1.583	1.051	2.385
	5th-minute Apgar	0.723	0.093	60.405	0.000	2.060	1.717	2.471
	5th -minute Apgar category	−0.787	0.220	12.842	0.000	0.455	0.296	0.700
	Gender	−0.122	0.109	1.264	0.261	0.885	0.715	1.095
	Height	0.084	0.024	12.622	0.000	1.088	1.038	1.139
	Birthweight	0.000	0.000	2.568	0.109	1.000	1.000	1.001
	Birthweight category	0.294	0.149	3.877	0.049	1.341	1.001	1.797
	Birth rank order	−0.344	0.393	0.766	0.381	0.709	0.328	1.531
	Number of the babies	0.483	0.908	0.283	0.595	1.620	0.274	9.598

The leading causes of neonatal and postneonatal mortality, with some cases exhibiting overlapping conditions, were as follows:

• Prematurity (n=453)
• RDS (n=325)
• Congenital abnormalities (n=160)
• Infections (n=72)
• Asphyxia (n=17)
• MAS (n=9)
• Other causes (n=79) (Supplemental 3)

Neonatal and postneonatal deaths are influenced by various factors, with congenital abnormalities accounting for 31% of these fatalities. The distribution of congenital abnormalities associated with death includes:

• *Cardiovascular malformations* (n=75): hypoplastic left heart syndrome (n=22), septal defects (n=19), valve diseases (n=12), Tetralogy of Fallot (n=7), transposition of the great arteries (n=5), atrioventricular complete block (n=5), situs inversus (n=2), double outlet right ventricle, ectopia cordis (n=1), obstructive cardiomyopathy (n=1), and Scimitar syndrome (n=1).
• *Central nervous system malformations* (n=29)*:* open neural tube defects (n=19), hydrocephalus (n=6), Galen vein aneurysm (n=1), cerebellar hypoplasia (n=1), Dandy-Walker syndrome (n=1), Carpenter syndrome (n=1), microphthalmia (n=1), and anophthalmia (n=1).
• *Gastrointestinal malformations* (n=22): omphalocele (n=8), gastroschisis (n=4), anal atresia (n=3), ileal atresia (n=3), esophageal atresia (n=3), and ventral herniation defect (n=1).
• *Musculoskeletal malformations* (n=20): congenital diaphragmatic hernia (n=15), thanatophoric dysplasia (n=2), sirenomelia (n=1), achondroplasia (n=1), and mitochondrial myopathy (n=1).
• *Pulmonary malformations* (n=19): pulmonary hypoplasia (n=9) and pulmonary atresia (n=7).
• *Genitourinary malformations* (n=11): bilateral renal dysplasia (n=5), bilateral renal agenesis (n=2), exstrophy of the bladder (n=1), and ambiguous genitalia.
• *Chromosomal anomalies* (n=7): Trisomy 18 (n=3), Trisomy 21 (n=2), Trisomy 13 (n=1), and DiGeorge syndrome (n=1).

**Figure 5:**
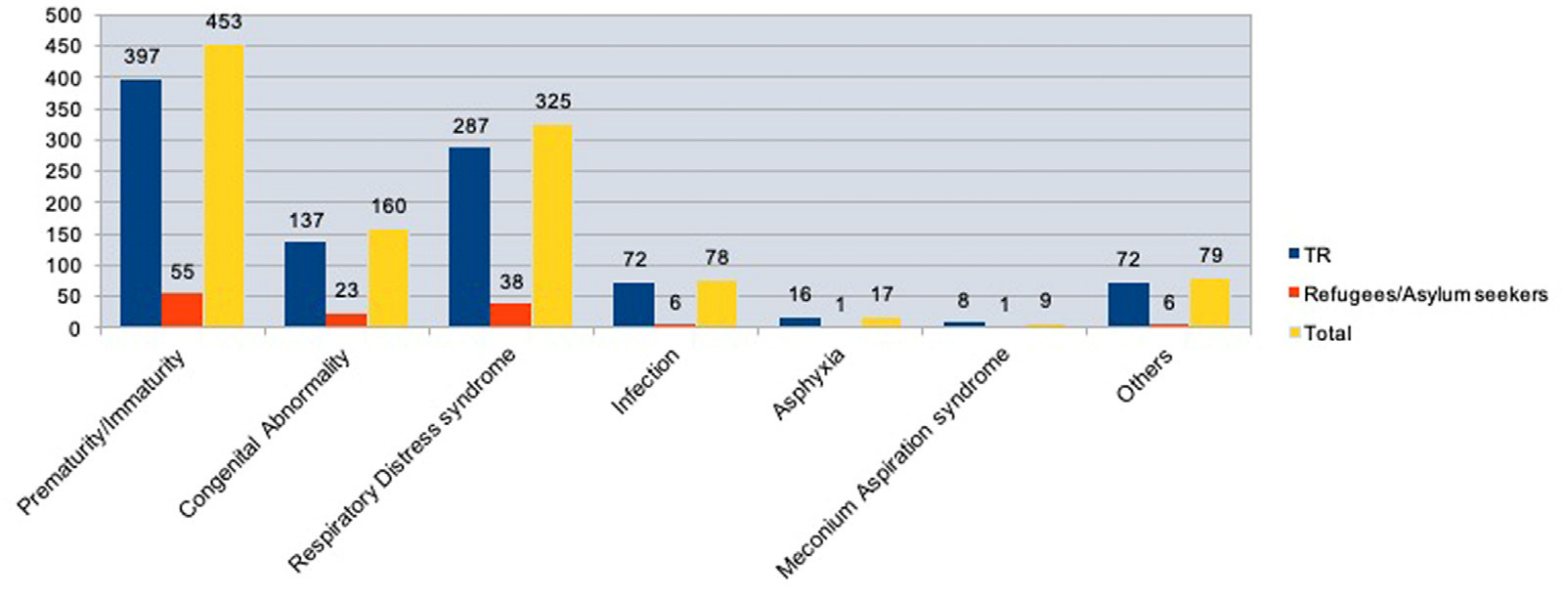
Distribution of the causes of neonatal/ postneonatal deaths in TR citizens, refugees/asylum seekers and total * More than one cause was identified. ^a^ X^2^ test revealed no significance. ** Others included metabolic diseases, hepatic failure, renal failure, heart failure, intestinal perforations, pneumotosis intestinalis, massive pulmonary embolism, gastrointestinal bleeding, multi-organ failure, sudden Infant death syndrome.

No significant difference was found in the frequency of congenital abnormalities between TR citizens (n=136) and refugees/asylum seekers (n=24) (*P*=.19) as the other causes leading to deaths (Figure 5).

Damla Çarkçı Yıldız. Association of Robson Ten Group Classification System with neonatal/postneonatal mortality. AJOG Glob Rep 2024.

## Comment

### Principle findings

Reports on CS rates are indistinct. The WHO Multi-country C-survey reported a CS rate of 17.9% in 42,361 live births, with intrapartum-related perinatal mortality at 3.0 per 1000 and perinatal mortality at 6.5 per 1000 live births for low-risk women.^16^ This study reports neonatal/postneonatal mortality rates for each Robson group in a tertiary referral center with a CS rate of 48.43%, including 40% repeat CSs.

#### Impact of high CS rates

This study contributes to the ongoing discussion regarding CS rates exceeding 19%, suggesting that such rates do not reduce neonatal mortality,^16^^,^^17^ though presented rates were from a tertiary referral center. This is a critical finding that challenges assumptions about the benefits of CSs.^18^^,^^19^

#### Risk factors for mortality

The analysis revealed that 89.08% of neonatal and postneonatal deaths were associated with:

• Abnormal fetal presentations (Groups R6, R7, R9),
• Multiple pregnancies (Group R8),
• Preterm births (Group R10).

These factors emerged as significant risk factors for neonatal and postneonatal mortality, aligning with previous literature suggesting that complications tied to prematurity, abnormal presentation, and low 5th-minute Apgar score are major contributors to mortality.^20^, ^21^, ^22^, ^23^, ^24^

#### Optimal CS rate and neonatal/postneonatal outcomes

• The study emphasizes that the relationship between the CS rate and optimal neonatal/postneonatal outcomes is complex. Various factors may influence outcomes, including:
  ○ Demographic, socioeconomic, and cultural factors,
  ○ Antenatal, intrapartum, and neonatal care,
  ○ The presence of congenital abnormalities,
  ○ Preterm birth rates,
  ○ Obstetric practices, and
  ○ Management of congenital anomalies in the antenatal, neonatal and postneonatal period.

Integrating the Robson TGCS with neonatal and postneonatal surveys could serve as a monitoring tool to assess the status of different institutions, identify risk factors, and track progress on a national level. This integrated approach is seen as more promising than simply tracking infant mortality or the CS rates.

#### Educational support and elimination of health disparities

• A key finding was the comparison between TR citizens and refugees/asylum seekers:
  • Refugees and asylum seekers were generally younger, had higher gravidity and parity, and delivered at lower gestational ages.
  • They also faced educational disadvantages, with a significant portion of Syrian refugees being illiterate or self-taught.^13^^,^^14^^,^^25^
• Despite these challenges, equitable access to antenatal, intrapartum, postpartum, and neonatal care, along with translation services and educational support, appears to have mitigated disparities in neonatal/ postneonatal mortality among refugee and asylum-seeker populations.

#### Maternal demographic factors

• Maternal demographics were not found to be significant risk factors in binary logistic regression analysis.
• This highlights the importance of focusing on clinical factors (such as fetal presentation, Apgar scores, and birth weight) rather than demographic factors alone when assessing neonatal outcomes.

### Results in the context of what is known

Integrating R TGCS with neonatal/postneonatal mortality appears to be rational as the first 28 days of life is the most vulnerable time for a child's survival.^20^ Neonatal mortality accounts for approximately two-thirds of the 8 million deaths in children less than 1 year of age, and nearly four-tenths of all deaths in children less than 5 years of age.^26^ Turkish Health Statistics reported that babies face the highest risk of dying within their first month of life, with a global neonatal mortality rate of 18 deaths per 1000 live births in 2021; a 51% decline from 37 deaths per 1000 live births in 1990.^24^

In our study, twins and higher order pregnancies, significantly contributed to neonatal and postneonatal mortality. These risks were primarily attributed to preterm delivery and low birth weight.^26^^,^^27^ Notably, 60% of twin pregnancies result in spontaneous labor before 37 weeks,^26^ and 12% occur before 32 weeks.^27^^,^^28^ Early onset pre-eclampsia and pregnancy-induced hypertension also develop in twins, with the risk being approximately 2- and 3-fold higher, respectively.^29^^,^^30^ The risk increases even further as the number of fetuses increases.^30^ Despite the recommendation to administer aspirin as early as 12 weeks of gestation until birth for women with multifetal pregnancies,^31^, ^32^, ^33^, ^34^ the quality of evidence supporting its use for preeclampsia prevention remains relatively low.^35^ While significant reduction in the risk of preeclampsia (RR, 0.67; 95% CI, 0.48−0.94) and mild preeclampsia (RR, 0.44; 95% CI, 0.24−0.82) were documented, this was not valid for severe preeclampsia (RR, 1.02; 95% CI, 0.61−1.72).

Yet, these are not the only challenges faced by twins and higher-order multiple pregnancies. Conditions specific to mono-chorionic multiple pregnancies, such as Twin-to-Twin Transfusion Syndrome, Twin Reversed Arterial Perfusion sequence, and Twin Anemia-Polycythemia Sequence, along with congenital abnormalities, umbilical cord complications, especially in monochorionic-monoamniotic pregnancies, are significant concerns. Additionally, iatrogenic complications and preterm premature rupture of membranes arising from invasive procedures (e.g., laser ablation, cord coagulation, radiofrequency ablation) also contributed to perinatal deaths.^36^

### Clinical implications and strengths of the study

• The study serves as an indicator of the healthcare services, translation support, and educational programs provided to refugees and asylum seekers in Türkiye.
• The potential benefits of combining these 2 classification may include:

#### Clinical audits and quality improvement

• Hospitals can track CS trends and neonatal outcomes more systematically.
• Helps in identifying high-risk groups and adjusting perinatal care strategies accordingly.

#### Neonatal care enhancement

• Supports early intervention strategies for high-risk pregnancies (e.g., improved neonatal resuscitation for preterm births).
• Facilitates better resource allocation in neonatal intensive care units and monitorization of the postnatal treatment results of fetuses especially with cardiac defects and diaphragmatic hernias

#### Policy and health service planning

• Governments and healthcare institutions can use these findings to establish evidence-based guidelines for CSs and neonatal care.
• Benchmarks can be set for neonatal and postneonatal mortality targets within maternal healthcare programs.

#### Data-driven decision making

• Encourages the use of TGCS-linked neonatal mortality data as a performance indicator in obstetric and neonatal care.
• Can inform training programs for obstetricians/ perinatologists and neonatologists on managing preterm labor, abnormal presentations, multiple pregnancies, and fetuses with congenital abnormalities.

### Limitations of the study

• This study does not qualify as a nationwide survey, as it was conducted in a tertiary referral center where women with preterm labor, multiple pregnancies with complications, and severe fetal anomalies requiring specialized antenatal and postnatal care were referred. Consequently, neonatal/postneonatal mortality rates derived from CSs in this setting may be overestimated.
• The study exclusively analyzed CSs, excluding vaginal deliveries.
• The impact of the COVID-19 pandemic, including delays or avoidance of necessary medical care, likely influenced mortality rates. Since the study period encompassed the outbreak and pandemic phase, excluding the first 3 months, the results should be interpreted with caution.
• Autopsy and genetic evaluations were conducted based on parental consent. However, the success rate of obtaining genetic results was significantly lower during the COVID-19 pandemic, leading to suboptimal genetic and pathological assessments.
• Neonatal and postneonatal deaths were not directly correlated with pregnancy complications, except for preterm labor.
• Distinctions between elective and emergency CSs could not be made,
• Trial of Labor (TOLAC) after CS was generally not practiced.
• Maternal complications associated with CSs were beyond the scope of this study.

## Conclusion

The integration of the R TGCS with neonatal and postneonatal mortality assessments offers a structured and standardized framework for evaluating obstetric and perinatal outcomes. Our findings underscore significant disparities in mortality rates across R TGCS, with the highest risk identified among multiple pregnancies, abnormal fetal presentations, and preterm cephalic presentations. These results emphasize the need for targeted perinatal interventions and risk-based obstetric strategies to improve neonatal outcomes.

## CRediT authorship contribution statement

**Damla Çarkçı Yıldız:** Writing – review & editing, Writing – original draft, Visualization, Software, Investigation, Formal analysis. **Elif Gül Yapar Eyi:** Writing – review & editing, Writing – original draft, Validation, Supervision, Project administration, Methodology, Conceptualization.

## Appendix Supplementary materials

Image, application 1

Image, application 2

Image, application 3

## Footnotes

Supplementary material associated with this article can be found in the online version at doi:10.1016/j.xagr.2025.100464.
