# Supplementary material for: Association of Robson Ten Group Classification System with neonatal/postneonatal mortality: an analysis for the effect of the mass migration
Source: AJOG Glob Rep. 2025 Feb 21;5(2):100464. doi: 10.1016/j.xagr.2025.100464 (PMC11964533; doi:10.1016/j.xagr.2025.100464)
Supplement: Supplementary file 1 [file mmc1.docx]

**Table Supplement 3** : Causes of neonatal and postneonatal deaths according to R TGCS* ^a^

|  | | **Total** | | **Prematurity** | | **RDS** | | **Congenital abnormality** | | **Infection** | | **Asphyxia** | | **MAS** | | **Others**** | | |
| --- | --- | --- | --- | --- | --- | --- | --- | --- | --- | --- | --- | --- | --- | --- | --- | --- | --- | --- |
|  |  | **n** | **%** | **n** | **%** | **n** | **%** | **n** | **%** | **n** | **%** | **n** | **%** | **n** | **%** | **n** | **%** |  |
| 1 | Total | 16 | 3.13 | 0 | 0.00 | 10 | 3.09 | 12 | 7.55 | 2 | 2.56 | 2 | 11.76 | 2 | 22.22 | 2 | 2.56 |  |
|  | TR | 14 | 87.50 | 0 | 0.00 | 9 | 90.00 | 11 | 91.67 | 1 | 50.00 | 1 | 50.00 | 1 | 50.00 | 1 | 50.00 |  |
|  | Refugees/ asylum seekers | 2 | 12.50 | 0 | 0.00 | 1 | 10.00 | 1 | 8.33 | 1 | 50.00 | 1 | 50.00 | 1 | 50.00 | 1 | 50.00 |  |
| 2 | Total | 4 | .78 | 0 | 0.00 | 1 | .31 | 2 | 1.26 | 0 | 0.00 | 2 | 11.76 | 0 | 0.00 | 0 | 0.00 |  |
|  | TR | 3 | 75.00 | 0 | 0.00 | 1 | 100.00 | 1 | 50.00 | 0 | 0.00 | 2 | 100.00 | 0 | 0.00 | 0 | 0.00 |  |
|  | Refugees/ asylum seekers | 1 | 25. 00 | 0 | 0.00 | 0 | 0.00 | 1 | 50.00 | 0 | 0.00 | 0 | 0.00 | 0 | 0.00 | 0 | 0.00 |  |
| 3 | Total | 10 | 1.95 | 1 | .22 | 2 | .62 | 10 | 6.29 | 1 | 1.28 | 0 | 0.00 | 0 | 0.00 | 1 | 1.28 |  |
|  | TR | 9 | 90.00 | 0 | 0.00 | 1 | 50.00 | 9 | 90.00 | 0 | 0.00 | 0 | 0.00 | 0 | 0.00 | 0 | 0.00 |  |
|  | Refugees/ asylum seekers | 1 | 10.00 | 1 | 100.00 | 1 | 50,00 | 1 | 10,00 | 1 | 100.00 | 0 | 0.00 | 0 | 0.00 | 1 | 100.00 |  |
| 4 | Total | 2 | .39 | 0 | 0.00 | 0 | 0,00 | 2 | 1.26 | 0 | 0.00 | 0 | 0.00 | 0 | 0.00 | 0 | 0.00 |  |
|  | TR | 2 | 100.00 | 0 | 0.00 | 0 | 0.00 | 2 | 100.00 | 0 | 0.00 | 0 | 0.00 | 0 | 0.00 | 0 | 0.00 |  |
|  | Refugees/ asylum seekers | 0 | .00 | 0 | 0.00 | 0 | 0.00 | 0 | 0.00 | 0 | 0.00 | 0 | 0.00 | 0 | 0.00 | 0 | 0.00 |  |
| 5 | Total | 24 | 4.69 | 2 | .44 | 15 | 4.63 | 17 | 10.69 | 3 | 3.85 | 0 | 0.00 | 2 | 22.22 | 4 | 3.85 |  |
|  | TR | 19 | 79.17 | 2 | 100.00 | 11 | 73.33 | 13 | 76.47 | 3 | 100.00 | 0 | 0.00 | 2 | 100.00 | 4 | 100.00 |  |
|  | Refugees/ asylum seekers | 5 | 20.83 | 0 | 0.00 | 4 | 26.67 | 4 | 23.53 | 0 | 0.00 | 0 | 0.00 | 0 | 0.00 | 0 | 0.00 |  |
| 6 | Total | 41 | 8.01 | 39 | 8.65 | 29 | 8.95 | 12 | 7.55 | 7 | 8.97 | 0 | 0.00 | 1 | 11.11 | 7 | 8.97 |  |
|  | TR | 38 | 92.68 | 36 | 92.31 | 26 | 89.66 | 11 | 91.67 | 6 | 85.71 | 0 | 0.00 | 1 | 100.00 | 6 | 85.71 |  |
|  | Refugees/ asylum seekers | 3 | 7.32 | 3 | 7.69 | 3 | 10.34 | 1 | 8.33 | 1 | 14.29 | 0 | 0.00 | 0 | 0.00 | 1 | 14.29 |  |
| 7 | Total | 46 | 8.98 | 40 | 8.87 | 27 | 8.33 | 12 | 7.55 | 7 | 8.97 | 0 | 0.00 | 1 | 11.11 | 7 | 8.97 |  |
|  | TR | 39 | 84.78 | 33 | 82.50 | 23 | 85.19 | 10 | 83.33 | 6 | 85.71 | 0 | 0.00 | 1 | 100.00 | 6 | 85.71 |  |
|  | Refugees/ asylum seekers | 7 | 15.22 | 7 | 17.50 | 4 | 14.81 | 2 | 16.67 | 1 | 14.29 | 0 | 0.00 | 0 | 0.00 | 1 | 14.29 |  |
| 8 | Total | 122 | 23.83 | 122 | 27.05 | 80 | 24.69 | 18 | 11.32 | 22 | 28.21 | 2 | 11.76 | 0 | 0.00 | 22 | 28.21 |  |
|  | TR | 107 | 87.70 | 107 | 87.70 | 72 | 90,00 | 15 | 83.33 | 21 | 95.45 | 2 | 100.00 | 0 | 0.00 | 21 | 95.45 |  |
|  | Refugees/asylum seekers | 15 | 12.30 | 15 | 12.30 | 8 | 10.00 | 3 | 16,67 | 1 | 4.55 | 0 | 0.00 | 0 | 0.00 | 1 | 4.55 |  |
| 9 | Total | 27 | 5.27 | 27 | 5.76 | 20 | 6.17 | 6 | 3.77 | 2 | 2.56 | 0 | 0.00 | 0 | 0.00 | 2 | 2.56 |  |
|  | TR | 23 | 85.19 | 23 | 88.46 | 17 | 85.00 | 5 | 83.33 | 2 | 100.00 | 0 | 0.00 | 0 | 0.00 | 2 | 100.00 |  |
|  | Refugees/asylum seekers | 4 | 14.81 | 4 | 11.54 | 3 | 15.00 | 1 | 16.67 | 0 | 0.00 | 0 | 0.00 | 0 | 0.00 | 0 | 0.00 |  |
| 10 | Total | 221 | 42.97 | 221 | 48.78 | 140 | 43.21 | 68 | 42.77 | 34 | 43.59 | 11 | 64.71 | 3 | 33.33 | 34 | 43.59 |  |
|  | TR | 195 | 88.18 | 195 | 88.18 | 126 | 90.00 | 59 | 86.76 | 33 | 97.06 | 11 | 100.00 | 3 | 100.00 | 33 | 97.06 |  |
|  | Refugees/ asylum seekers | 26 | 11,82 | 26 | 11.82 | 14 | 10.00 | 9 | 13.24 | 1 | 2.94 | 0 | 0.00 | 0 | 0.00 | 1 | 2.94 |  |
| Total | | 513 | 100 | 452 | 88.11 | 325 | 63.35 | 160 | 31.19 | 78 | 15.20 | 17 | 3.31 | 9 | 1.75 | 79 | 15.40 |  |

* More than one cause was identified ^a^ Chi-square test

** Others included metabolic diseases, hepatic failure, renal failure, heart failure, intestinal perforations, pneumotosis intestinalis, massive pulmonary

embolism, gastrointestinal bleeding, multiorgan failure, Sudden Infant Death Syndrome
