# Supplementary material for: Association of Robson Ten Group Classification System with neonatal/postneonatal mortality: an analysis for the effect of the mass migration
Source: AJOG Glob Rep. 2025 Feb 21;5(2):100464. doi: 10.1016/j.xagr.2025.100464 (PMC11964533; doi:10.1016/j.xagr.2025.100464)
Supplement: Supplementary file 2 [file mmc2.docx]

**Table Supplement 1.** APGAR scores, presence of neonatal unit admission, duration of hospital stay and presence of death according to Robson

classification and nationality of CS births

|  | | **APGAR 1^st^ minute^a^** | | | | | | | **APGAR 5^th^ minute^a^** | | | | | | | | **Neonatal Unit admission^a^** | | **Duration of hospital**  **stay^b-c^ (days)** | | **Presence of neonatal/**  **post neonatal death^a^** | | **Total** | |
| --- | --- | --- | --- | --- | --- | --- | --- | --- | --- | --- | --- | --- | --- | --- | --- | --- | --- | --- | --- | --- | --- | --- | --- | --- |
|  |  | **<4** | | **4-6** | | | **≥7** | | **<4** | | | **4-6** | | | **≥7** | |  |  |  |  |  |  |  |  |
|  |  | **n** | **%** | **n** | **%** | **n** | | **%** | **n** | **%** | **n** | | **%** | **n** | | **%** | **n** | **%** | **Mean + SD** | **n** | | (‰) | **n** | **%** |
| 1 | Total | 28 | 0.9 | 178 | 5.8 | 2856 | | 93.3 | 6 | 0.2 | 35 | | 1.1 | 3021 | | 98.7 | 411 | 13.4 | 9.31±12.60 | 16 | | 5.22 | 3062 | 11.6 |
|  | TR | 26 | 0.9 | 156 | 5.6 | 2617 | | 93.5 | 6 | 0.2 | 33 | | 1.2 | 2760 | | 98.6 | 394 | 14.1 | 9.32±12.79 | 14 | | 5.00 | 2799 | 91.4 |
|  | Refugees/asylum seekers | 2 | 0.8 | 22 | 8.4 | 239 | | 90.9 | 0 | 0.0 | 2 | | 0.8 | 261 | | 99.2 | 17 | 6.5 | 9.29±7.37 | 2 | | 7.60 | 263 | 8.6 |
| 2 | Total | 7 | 0.2 | 114 | 4.0 | 2708 | | 95.7 | 1 | 0.0 | 12 | | 0.4 | 2816 | | 99.5 | 306 | 10.8 | 5.88±7.65 | 4 | | 1.41 | 2829 | 10.7 |
|  | TR | 7 | 0.3 | 100 | 3.8 | 2496 | | 95.9 | 1 | 0.0 | 12 | | 0.5 | 2590 | | 99.5 | 293 | 11.3 | 5.91±7.79 | 3 | | 1.15 | 2603 | 92.0 |
|  | Refugees/ asylum seekers | 0 | 0.0 | 14 | 6.2 | 212 | | 93.8 | 0 | 0.0 | 0 | | 0.0 | 226 | | 100.0 | 13 | 5.8 | 5.15±3.31 | 1 | | 4.42 | 226 | 8.0 |
| 3 | Total | 21 | 1.6 | 106 | 8.0 | 1204 | | 90.5 | 3 | 0.2 | 27 | | 2.0 | 1301 | | 97.7 | 186 | 13.9 | 14.71±25.31 | 10 | | 7.51 | 1331 | 5.0 |
|  | TR | 17 | 1.4 | 89 | 7.5 | 1077 | | 91.0 | 1 | 0.1 | 23 | | 1.9 | 1159 | | 98.0 | 164 | 13.9 | 14.74±25.03 | 9 | | 7.60 | 1183 | 88.9 |
|  | Refugees/asylum seekers | 4 | 2.7 | 17 | 11.5 | 127 | | 85.8 | 2 | 1.4 | 4 | | 2.7 | 142 | | 95.9 | 22 | 14.9 | 14.50±27.94 | 1 | | 6.75 | 148 | 11.1 |
| 4 | Total | 2 | 0.2 | 58 | 6.4 | 851 | | 93.4 | 0 | 0.0 | 5 | | 0.5 | 906 | | 99.5 | 99 | 10.9 | 9.92±19.22 | 2 | | 2.19 | 911 | 3.4 |
|  | TR | 2 | 0.3 | 45 | 5.7 | 748 | | 94.1 | 0 | 0.0 | 4 | | 0.5 | 791 | | 99.5 | 78 | 9.8 | 9.81±20.91 | 2 | | 2.51 | 795 | 87.3 |
|  | Refugees/asylum seekers | 0 | 0.0 | 13 | 11.2 | 103 | | 88.8 | 0 | 0.0 | 1 | | 0.9 | 115 | | 99.1 | 21 | 18.1 | 10.33±11.28 | 0 | | - | 116 | 12.7 |
| 5 | Total | 18 | 0.2 | 261 | 2.5 | 9968 | | 97.3 | 9 | 0.1 | 19 | | 0.2 | 10219 | | 99.7 | 951 | 9.3 | 7.96±13.63 | 24 | | 2.34 | 10247 | 38.7 |
|  | TR | 17 | 0.2 | 208 | 2.3 | 8814 | | 97.5 | 9 | 0.1 | 16 | | 0.2 | 9014 | | 99.7 | 828 | 9.2 | 7.95±13.82 | 19 | | 2.10 | 9039 | 88.2 |
|  | Refugees/asylum seekers | 1 | 0.1 | 53 | 4.4 | 1154 | | 95.5 | 0 | 0.0 | 3 | | 0.2 | 1205 | | 99.8 | 123 | 10.2 | 8.02±12.32 | 5 | | 4.13 | 1208 | 11.8 |
| 6 | Total | 40 | 4.3 | 146 | 15.8 | 737 | | 79.8 | 12 | 1.3 | 44 | | 4.8 | 867 | | 93.9 | 223 | 24.2 | 27.53±36.74 | 41 | | 44.42 | 923 | 3.5 |
|  | TR | 37 | 4.4 | 131 | 15.5 | 677 | | 80.1 | 12 | 1.4 | 40 | | 4.7 | 793 | | 93.8 | 214 | 25.3 | 26.72±35.31 | 38 | | 44.97 | 845 | 91.5 |
|  | Refugees/ asylum seekers | 3 | 3.8 | 15 | 19.2 | 60 | | 76.9 | 0 | 0.0 | 4 | | 5.1 | 74 | | 94.9 | 9 | 11.5 | 46.78±61.84 | 3 | | 38.46 | 78 | 8,5 |
| 7 | Total | 60 | 5.4 | 216 | 19.5 | 829 | | 75.0 | 14 | 1.3 | 70 | | 6.3 | 1021 | | 92.4 | 318 | 28.8 | 23.86±27.79 | 46 | | 41,63 | 1105 | 4,2 |
|  | TR | 49 | 5.1 | 189 | 19.7 | 719 | | 75.1 | 11 | 1.1 | 60 | | 6.3 | 886 | | 92.6 | 280 | 29.3 | 23.89±27.03 | 39 | | 40.75 | 957 | 86.6 |
|  | Refugees/ asylum seekers | 11 | 7.4 | 27 | 18.2 | 110 | | 74.3 | 3 | 2.0 | 10 | | 6.8 | 135 | | 91.2 | 38 | 25.7 | 23.61±33.33 | 7 | | 47.29 | 148 | 13.4 |
| 8 | Total* | 107 | 6.2 | 675 | 39.3 | 937 | | 54.5 | 18 | 1.0 | 161 | | 9.4 | 1540 | | 89.6 | 932 | 54.2 | 26.23±31.98 | 122 | | 70.97 | 1719 | 6.5 |
|  | TR | 93 | 6.1 | 595 | 39.1 | 834 | | 54.8 | 13 | 0.9 | 143 | | 9.4 | 1366 | | 89.8 | 842 | 55.3 | 26.71±32.52 | 107 | | 70.30 | 1522 | 88.5 |
|  | Refugees/ asylum seekers | 14 | 7.1 | 80 | 40.6 | 103 | | 52.3 | 5 | 2,5 | 18 | | 9,1 | 174 | | 88,3 | 90 | 45,7 | 21,77±26,15 | 15 | | 76.14 | 197 | 11,5 |
| 9 | Total | 30 | 8.0 | 89 | 23.7 | 257 | | 68.4 | 7 | 1.9 | 38 | | 10.1 | 331 | | 88.0 | 143 | 38.0 | 28.47±37.46 | 27 | | 71.81 | 376 | 1.4 |
|  | TR | 26 | 7.8 | 77 | 23.1 | 231 | | 69.2 | 5 | 1.5 | 34 | | 10.2 | 295 | | 88.3 | 133 | 39.8 | 29.29±38.26 | 23 | | 68.86 | 334 | 88.8 |
|  | Refugees/ sylum seekers | 4 | 9.5 | 12 | 28.6 | 26 | | 61.9 | 2 | 4.8 | 4 | | 9.5 | 36 | | 85.7 | 10 | 23.8 | 17.50±22.83 | 4 | | 95.23 | 42 | 11.2 |
| 10 | Total | 256 | 6.4 | 965 | 24.3 | 2750 | | 69.3 | 88 | 2.2 | 279 | | 7.0 | 3604 | | 90.8 | 1974 | 49.7 | 21.57±30.11 | 221 | | 55.65 | 3971 | 15.0 |
|  | TR | 231 | 6.6 | 849 | 24.4 | 2401 | | 69.0 | 79 | 2.3 | 250 | | 7.2 | 3152 | | 90.5 | 1784 | 51.2 | 21.50±29.80 | 195 | | 56.01 | 3481 | 87.7 |
|  | Refugees/ asylum seekers | 25 | 5.1 | 116 | 23.7 | 349 | | 71.2 | 9 | 1.8 | 29 | | 5.9 | 452 | | 92.2 | 190 | 38. 8 | 22.23±32.98 | 26 | | 53.06 | 490 | 12.3 |
|  | Total | 569 | 2.2 | 2808 | 10.6 | 23097 | | 87.2 | 158 | 0.6 | 690 | | 2.6 | 25626 | | 96.8 | 5543 | 20.9 | 23.73±34.98 | 513 | | 19.37 | 26474 | 100.0 |
|  | TR | 505 | 2.1 | 2439 | 10.4 | 20614 | | 87.5 | 137 | 0.6 | 615 | | 2.6 | 22806 | | 96.8 | 5010 | 21.3 | 23.50±34.80 | 449 | | 19.05 | 23558 | 89.0 |
|  | Refugees/ asylum seekers | 64 | 2.2 | 369 | 12.6 | 2483 | | 85.2 | 21 | 0.7 | 75 | | 2.6 | 2820 | | 96.7 | 533 | 18.0 | 24.23±32.98 | 64 | | 21.94 | 2916 | 11.0 |

*64 death fetuses in twins and higher order pregnancies weighing>500 grams in R8 were not included in neonatal deaths

**^a^** Chi-square test

**^b^** Mann Whitney U test for comparison between two groups,

^c^ Kruskall Wallis analysis for comparison of more than two groups
