# Supplementary material for: Association of Robson Ten Group Classification System with neonatal/postneonatal mortality: an analysis for the effect of the mass migration
Source: AJOG Glob Rep. 2025 Feb 21;5(2):100464. doi: 10.1016/j.xagr.2025.100464 (PMC11964533; doi:10.1016/j.xagr.2025.100464)
Supplement: Supplementary file 3 [file mmc3.docx]

Table Supplement 2: Difference in neonatal and post-neonatal deaths according to Robson groups

| (I)  Robson | (J)  Robson | Mean Difference  (I-J) | SE | Sig. | 95% CI Interval | |
| --- | --- | --- | --- | --- | --- | --- |
|  |  |  |  |  | Lower Bound | Upper  Bound |
| **1** | 2 | -.009^*^ | .002 | .001 | -.02 | .00 |
|  | 3 | .002 | .004 | 1.000 | -.01 | .01 |
|  | 4 | -.006 | .003 | .703 | -.02 | .00 |
|  | 5 | -.006 | .002 | .092 | -.01 | .00 |
|  | 6 | .040^*^ | .007 | .000 | .02 | .06 |
|  | 7 | .032^*^ | .006 | .000 | .01 | .05 |
|  | 8 | .060^*^ | .006 | .000 | .04 | .08 |
|  | 9 | .066^*^ | .014 | .000 | .02 | .11 |
|  | 10 | .052^*^ | .004 | .000 | .04 | .07 |
| **2** | 1 | .009^*^ | .002 | .001 | .00 | .02 |
|  | 3 | .011^*^ | .003 | .039 | .00 | .02 |
|  | 4 | .002 | .002 | 1.000 | -.01 | .01 |
|  | 5 | .003 | .001 | .587 | .00 | .01 |
|  | 6 | .049^*^ | .007 | .000 | .03 | .07 |
|  | 7 | .040^*^ | .006 | .000 | .02 | .06 |
|  | 8 | .069^*^ | .006 | .000 | .05 | .09 |
|  | 9 | .075^*^ | .014 | .000 | .03 | .12 |
|  | 10 | .061^*^ | .004 | .000 | .05 | .07 |
| **3** | 1 | -.002 | .004 | 1.000 | -.01 | .01 |
|  | 2 | -.011^*^ | .003 | .039 | -.02 | .00 |
|  | 4 | -.008 | .004 | .704 | -.02 | .00 |
|  | 5 | -.008 | .003 | .375 | -.02 | .00 |
|  | 6 | .038^*^ | .008 | .000 | .01 | .06 |
|  | 7 | .030^*^ | .007 | .001 | .01 | .05 |
|  | 8 | .058^*^ | .007 | .000 | .04 | .08 |
|  | 9 | .064^*^ | .014 | .000 | .02 | .11 |
|  | 10 | .050^*^ | .005 | .000 | .03 | .07 |
| **4** | 1 | .006 | .003 | .703 | .00 | .02 |
|  | 2 | -.002 | .002 | 1.000 | -.01 | .01 |
|  | 3 | .008 | .004 | .704 | .00 | .02 |
|  | 5 | .000 | .002 | 1.000 | -.01 | .01 |
|  | 6 | .047^*^ | .008 | .000 | .02 | .07 |
|  | 7 | .038^*^ | .006 | .000 | .02 | .06 |
|  | 8 | .067^*^ | .007 | .000 | .05 | .09 |
|  | 9 | .073^*^ | .014 | .000 | .03 | .12 |
|  | 10 | .058^*^ | .004 | .000 | .04 | .07 |
| **5** | 1 | .006 | .002 | .092 | .00 | .01 |
|  | 2 | -.003 | .001 | .587 | -.01 | .00 |
|  | 3 | .008 | .003 | .375 | .00 | .02 |
|  | 4 | .000 | .002 | 1.000 | -.01 | .01 |
|  | 6 | .046^*^ | .007 | .000 | .02 | .07 |
|  | 7 | .038^*^ | .006 | .000 | .02 | .06 |
|  | 8 | .066^*^ | .006 | .000 | .05 | .09 |
|  | 9 | .072^*^ | .014 | .000 | .03 | .12 |
|  | 10 | .058^*^ | .004 | .000 | .05 | .07 |
| **6** | 1 | -.040^*^ | .007 | .000 | -.06 | -.02 |
|  | 2 | -.049^*^ | .007 | .000 | -.07 | -.03 |
|  | 3 | -.038^*^ | .008 | .000 | -.06 | -.01 |
|  | 4 | -.047^*^ | .008 | .000 | -.07 | -.02 |
|  | 5 | -.046^*^ | .007 | .000 | -.07 | -.02 |
|  | 7 | -.008 | .009 | 1.000 | -.04 | .02 |
|  | 8 | .020 | .010 | .803 | -.01 | .05 |
|  | 9 | .026 | .016 | .987 | -.02 | .08 |
|  | 10 | .012 | .008 | .999 | -.01 | .04 |
| **7** | 1 | -.032^*^ | .006 | .000 | -.05 | -.01 |
|  | 2 | -.040^*^ | .006 | .000 | -.06 | -.02 |
|  | 3 | -.030^*^ | .007 | .001 | -.05 | -.01 |
|  | 4 | -.038^*^ | .006 | .000 | -.06 | -.02 |
|  | 5 | -.038^*^ | .006 | .000 | -.06 | -.02 |
|  | 6 | .008 | .009 | 1.000 | -.02 | .04 |
|  | 8 | .028^*^ | .009 | .047 | .00 | .06 |
|  | 9 | .035 | .015 | .632 | -.01 | .08 |
|  | 10 | .020 | .007 | .204 | .00 | .04 |
| **8** | 1 | -.060^*^ | .006 | .000 | -.08 | -.04 |
|  | 2 | -.069^*^ | .006 | .000 | -.09 | -.05 |
|  | 3 | -.058^*^ | .007 | .000 | -.08 | -.04 |
|  | 4 | -.067^*^ | .007 | .000 | -.09 | -.05 |
|  | 5 | -.066^*^ | .006 | .000 | -.09 | -.05 |
|  | 6 | -.020 | .010 | .803 | -.05 | .01 |
|  | 7 | -.028^*^ | .009 | .047 | -.06 | .00 |
|  | 9 | .006 | .015 | 1.000 | -.04 | .06 |
|  | 10 | -.008 | .007 | 1.000 | -.03 | .02 |
| **9** | 1 | -.066^*^ | .014 | .000 | -.11 | -.02 |
|  | 2 | -.075^*^ | .014 | .000 | -.12 | -.03 |
|  | 3 | -.064^*^ | .014 | .000 | -.11 | -.02 |
|  | 4 | -.073^*^ | .014 | .000 | -.12 | -.03 |
|  | 5 | -.072^*^ | .014 | .000 | -.12 | -.03 |
|  | 6 | -.026 | .016 | .987 | -.08 | .02 |
|  | 7 | -.035 | .015 | .632 | -.08 | .01 |
|  | 8 | -.006 | .015 | 1.000 | -.06 | .04 |
|  | 10 | -.014 | .014 | 1.000 | -.06 | .03 |
| **10** | 1 | -.052^*^ | .004 | .000 | -.07 | -.04 |
|  | 2 | -.061^*^ | .004 | .000 | -.07 | -.05 |
|  | 3 | -.050^*^ | .005 | .000 | -.07 | -.03 |
|  | 4 | -.058^*^ | .004 | .000 | -.07 | -.04 |
|  | 5 | -.058^*^ | .004 | .000 | -.07 | -.05 |
|  | 6 | -.012 | .008 | .999 | -.04 | .01 |
|  | 7 | -.020 | .007 | .204 | -.04 | .00 |
|  | 8 | .008 | .007 | 1.000 | -.02 | .03 |
|  | 9 | .014 | .014 | 1.000 | -.03 | .06 |
| *. The mean difference is significant at the 0.05 level. | | | | | | |
